# Current Best Practices for Analysis of Dendritic Spine Morphology and Number in Neurodevelopmental Disorder Research

**DOI:** 10.1021/acschemneuro.3c00062

**Published:** 2023-04-18

**Authors:** Ben-Zheng Li, Anna Sumera, Sam A Booker, Elizabeth A. McCullagh

**Affiliations:** † Department of Integrative Biology, Oklahoma State University, Stillwater, Oklahoma 74078, United States; ‡ Simons Initiative for the Developing Brain, Centre for Discovery Brain Sciences, University of Edinburgh, Edinburgh EH8 9XD, U.K.; § Department of Physiology and Biophysics, University of Colorado Anschutz Medical Campus, Aurora, Colorado 80045, United States

**Keywords:** neural anatomy, dendritic spine, synapse, neural plasticity, spine morphology, neurodevelopmental disorder

## Abstract

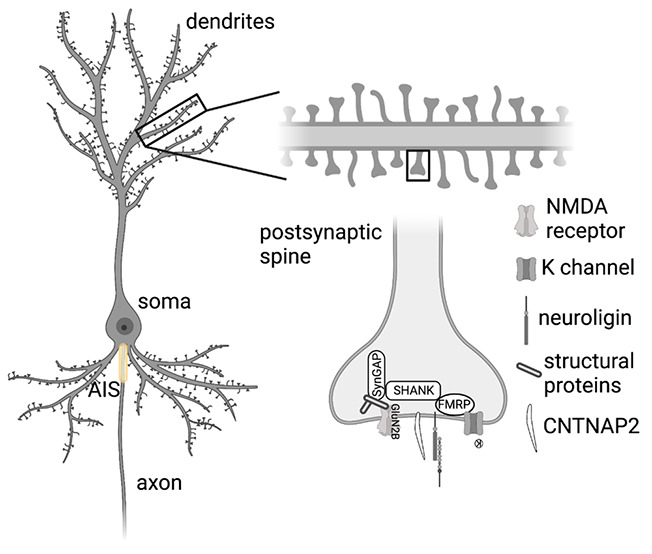

Quantitative methods for assessing neural anatomy have rapidly evolved in neuroscience and provide important insights into brain health and function. However, as new techniques develop, it is not always clear when and how each may be used to answer specific scientific questions posed. Dendritic spines, which are often indicative of synapse formation and neural plasticity, have been implicated across many brain regions in neurodevelopmental disorders as a marker for neural changes reflecting neural dysfunction or alterations. In this Perspective we highlight several techniques for staining, imaging, and quantifying dendritic spines as well as provide a framework for avoiding potential issues related to pseudoreplication. This framework illustrates how others may apply the most rigorous approaches. We consider the cost-benefit analysis of the varied techniques, recognizing that the most sophisticated equipment may not always be necessary for answering some research questions. Together, we hope this piece will help researchers determine the best strategy toward using the ever-growing number of techniques available to determine neural changes underlying dendritic spine morphology in health and neurodevelopmental disorders.

## Introduction

Neurodevelopmental conditions can include a broad scope of disorders including rare diseases,^1^ schizophrenia,^2^ and disorders associated with autism, which affect up to 3% of the population worldwide.^3,4^ These conditions can co-occur with intellectual disability, sensory disturbances, altered social interactions, and epilepsy.^5^ The underlying cause of such conditions can be attributed to genetic, environmental, or idiopathic mechanisms.^6^ However, it is largely accepted that altered cell function during neurodevelopment is central to these changes in behavior. While some features may be corrected later in life with therapeutic intervention, ascertaining how neurodevelopmental modifiers lead to altered neuron function in establishing brain circuits remains key to developing more efficacious therapies and understanding these conditions.

**Figure 1:**
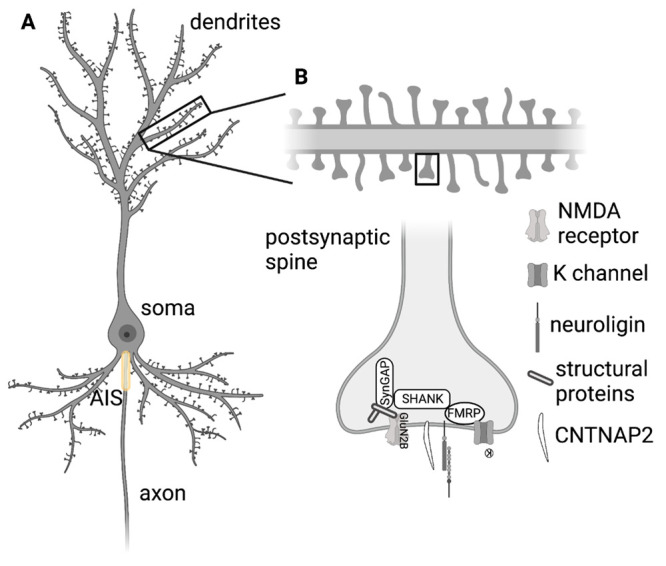
Structure of a neuron and relationship with neurodevelopmental disorders. (A) shows a pyramidal neuron with dendritic tree (top), soma, axon initial segment (AIS), and axon. (B) depicts a zoom-in on a single dendritic spine and some of the components including proteins relevant to genetic forms of ASD (Shank, FMRP, SynGAP, CNTNAP2) and some of the postsynaptic components to which they are associated (K channels, NMDA receptors, structural proteins, and neuroligins). Note that this diagram is not comprehensive in its depiction of the postsynaptic dendrite or all the known interactions with proteins important for neurodevelopmental disorders.

It is generally accepted that neurons are key integrative elements in brain circuits, comprising a dendritic arbor which receives the majority of synaptic inputs; a soma, which is a key integration point; an axon initial segment (AIS), which dictates action potential discharge; and an axon, which provides local and long-range output of neurons (see Figure 1A). Understanding the structure of these different cellular compartments and how they change over development gives us a detailed insight into how neurons function with respect to their inputs and their outputs. Indeed, in many genetic models of neurodevelopmental disorders, such changes in neuronal structure have been observed, such as reduced dendritic complexity,^7,8^ decreased or increased dendritic spine density,^9−12^ altered cell body size,^13^ AIS length,^14,15^ axonal complexity,^16^ or presynaptic axon terminal numbers.^17,18^ These and other changes in neuronal structure can have large-scale functional consequences for the activity of neurons and how they process information at the cellular and circuit level, which have been reviewed elsewhere.^19−22^ Dendritic spines, in particular, have an important role in synaptic plasticity including long-term potentiation and depression and can be considered an anatomical correlate to overall synaptic function.^22^

With this in mind, this Perspective article is going to focus on imaging of the somatodendritic compartment of neurons and how one may determine the dendritic structure and dendritic spine properties, looking at key technical and analytical considerations. Dendritic spines are of particular interest here, as many genes associated with neurodevelopmental impairments are involved in synaptic function. For example, based on the SFARI list of autism risk genes, many of the most penetrant genes have a role in synapse formation, stabilization, or function (https://gene.sfari.org/). Indeed, genes such as *SYNGAP, FMR1, GRIN2B*, and *SHANK3* give rise to proteins that are highly expressed at postsynaptic membranes, serving important roles in their structure and function (Figure 1B). In addition to genes specifically associated with autism, synaptic genes, such as *GRIN2A*,^23^*GRIA3*,^24^*NRXN1*,^25^ are also of interest in schizophrenia and similarly have important roles in synapse function and development.^26^ After 10 days of life in rodents, the majority of excitatory synapses on principal cells are localized to dendritic spines;^27^ this structure may give an approximation for the number of glutamatergic synaptic contacts. For this reason, measurements of spine density (and shape) have become ubiquitous in neurodevelopmental research.^28^ However, there are many methods and conceptual considerations that need to be accounted for when determining these features. This article aims to set out the current best practices for measuring dendritic spine density and morphology in development, their applicability to other neuronal structures, and the key considerations that should be made when performing these analyses. We hope to provide a roadmap for the appropriate measurement of neuronal structures that is applicable to the widest possible cohort of researchers around the world. Indeed, while this Perspective focuses on neurodevelopmental disorders, dendritic spine changes are also important for learning,^29^ drug administration,^30^ and a variety of pathological states including neurodegenerative disorders such as Alzheimer’s disease and dementia,^31,32^ Huntington’s disease,^33^ stroke,^34^ and aging,^35^ emphasizing the importance of spines and broad relevance of this topic.

## How Do You Decide What Technique Is Appropriate for Your Biological Question, and What Can You Hope To Achieve?

While imaging neuronal spines has become an important method for better understanding neural function, recent advances in technology have made these experiments more feasible and less time-consuming. There are many considerations to be made in spine imaging including how cells/spines are labeled, imaging platform, and analysis pipeline. With new technologies ever emerging, there are many options that may be appropriate based on the scientific questions posed that help determine labeling, imaging, and analysis, particularly when funding and costs for many techniques may be prohibitive.

**Figure 2:**
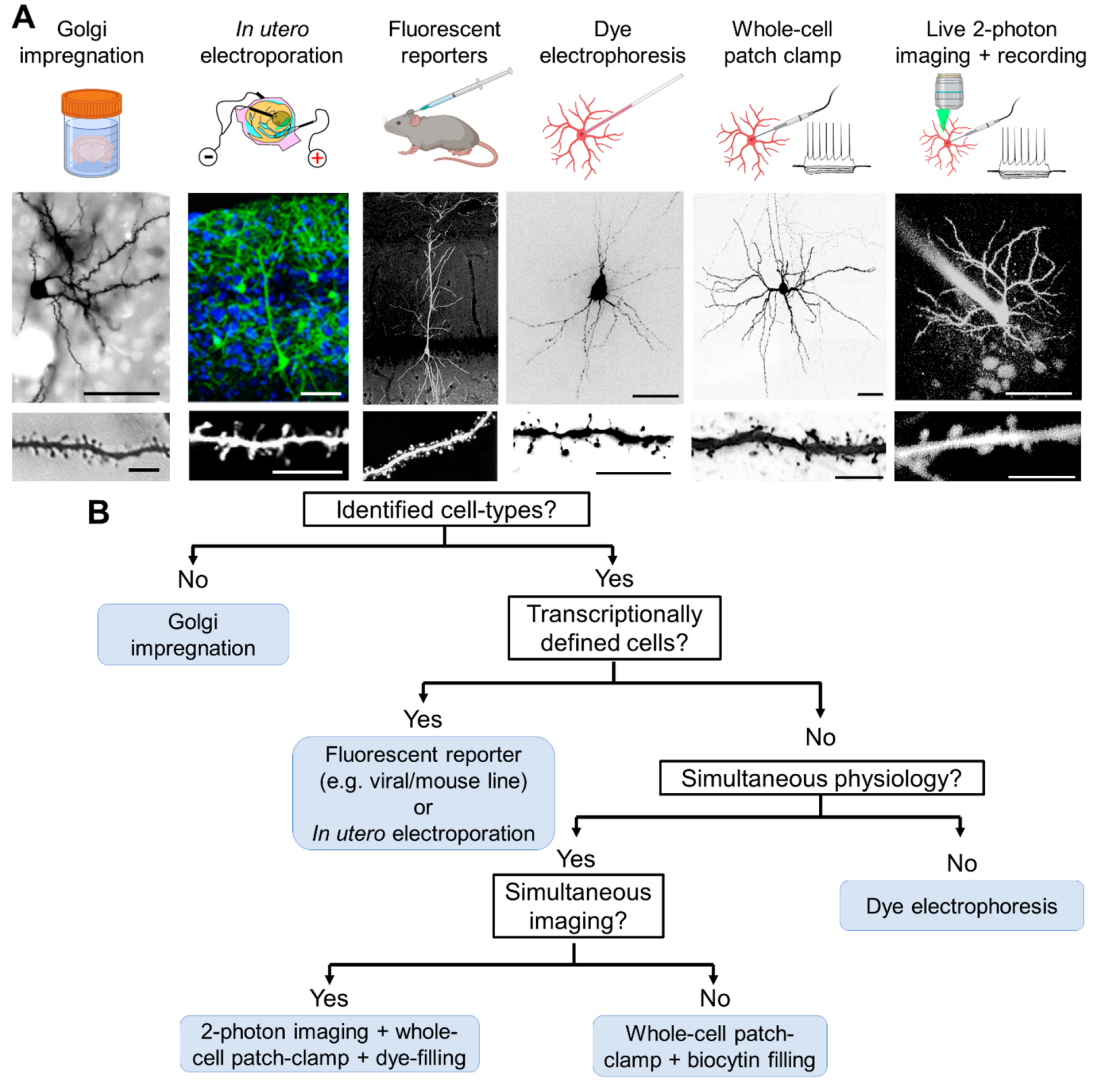
Summary of key methods to label neurons in either fixed or living brain tissue. (A) Overview of key experimental approaches to label cells in fixed tissues (with or without reporters) or labeling of cells in brain tissue. Below each methodology, example cells that have been recovered from each method are shown. Scale bars: 50 and 10 μm. (B) Decision tree of how to determine which method may be most applicable to a given scientific question. Note that several arms of this tree may in fact be applicable to other aspects too. Figure elements are adapted from refs (2), (41), (55), and (62) with permission provided by *Neuron*;^41^CC-BY-4.0,^2,55^ Copyright 2008 Society for Neuroscience,^62^ or unpublished data (S. Booker/A. Sumera).

The initial decision of what dyes/labeling method to use is largely dependent on the type of scientific question posed and/or the resources available to answer that question (Figure 2). The classic approach taken by notable pioneers in the field was the use of Golgi impregnation.^36,37^ While little has changed in the technique over the following century, this approach still yields high-quality structural stains of neurons in fixed tissues.^38^ However, this method does not allow for selective labeling of a predetermined cell type. Given the rise in transcriptomic identity of different cell types in rodent and human brains,^39,40^ one of the most high-yield approaches is to label cells based on gene expression. Particularly, if one uses a Cre-recombinase expressed under a specific gene promoter, either viral or off-the-shelf reporter animal lines can be employed to target fluorescent protein expression (e.g., green, yellow, or red fluorescent proteins {GFP, YFP, RFP}) in order to bulk-label many cells expressing that gene.^41^ A simpler incarnation of this approach, which has less cell-type specificity, is to use fluorescent reporters driven by a *Thy-*1 promoter, which through random genome insertion leads to a variety of different cell types being labeled. Similar to genetically encoded or viral labeling approaches, *in vivo* or *in utero* electroporation can be used to sparsely label genetically identified cell populations based on the expression of genetic markers and their localization within the brain,^2,42,43^^44^ thus overcoming the necessity for specifically breeding animal lines expressing fluorescent reporters, which may not be readily available. This technique however requires significant surgical expertise to accurately label the desired populations of neurons based on their precise location within the developing brain. These labeled cells can then be classified based on overall cell structure or immunohistochemical identification prior to spine measurements,^45^ allowing for *post-hoc* identification of neuronal subtypes or a more detailed characterization of spine structure and function.^46,47^ If such approaches are not possible, due to reduced ability to cross-breed specific transgenic rodent lines, one approach is to perform electrophoretic dye-filling of neurons from fixed brain tissue.^48^ By using ionically charged dyes, single cells in lightly fixed brain tissue can be impaled with sharp electrodes. Once inside a cell, this electrode is exposed to a pulse-train of voltage stimuli, which drives movement of the dye into the cytoplasm of the impaled cell. Once multiple cells from the same slice have been filled in this manner, the tissue section can then be postfixed, mounted on a slide, and imaged immediately or further processed with immunohistochemistry. This approach is therefore relatively high throughput, although the quality of filling is often dependent on tissue quality and fixation time, meaning that it may not always be the most appropriate method for investigating dendritic spine morphology due to incomplete fills. Other alternative and low-cost methods of visualizing dendritic spines include DiI microcrystal labeling^49,50^ and gene-gun DiOlistic labeling.^51^ Due to the similar labeling pattern and nonspecific nature of these approaches, they can be viewed as equivalent to Golgi impregnation.

Arguably the most powerful approach to labeling of neurons combines dye-filling with concomitant electrophysiological recording. The use of whole-cell patch-clamp recordings when the recording electrode is filled with either biocytin or a fluorescent morphometric dye allows for the intrinsic electrical and synaptic activity of a living cell to be measured and labeled simultaneously.^52,53^ This approach utilizes the general impermeability of the plasma membrane of neurons to these exogenous labels and dyes, and allows for correlated physiology/morphology characterization of cell types. A recent development of this approach also allows the harvesting of cytoplasmic RNA for sequencing, to enable the correlation of the transcriptome with morphology and physiology.^54^ The most complete approach that combines physiology and morphology is to perform these simultaneously with the imaging modality of choice, e.g., two-photon. This allows the determination of dendritic spine dynamics in real time alongside a physiological assay. In our recent study, we show that from identified dendritic spines, despite typical spine number and structure, the function of individual spines is impaired in a mouse model of Fragile X Syndrome (*Fmr1* knockout mice).^55^ These data, however, somewhat are at odds with earlier studies in *Fmr1* knockout mice, where prominent changes in density and structure were observed,^56−58^ observations which have been reviewed previously.^28,59^ Although the live images of dendritic spines we generated using two-photon imaging were sufficient for determining spine density, they were not appropriate for the measurement of dendritic spine structure. Another well described alternative approach is to use *in vivo* multiphoton imaging to measure spine density and dynamics, including in models of neurodevelopmental disorders.^59−61^ While these approaches have the distinct advantage of occurring in the intact brain, a full summary of their *pros* and *cons* is beyond the remit of this current Perspective. The reasons that such live multiphoton imaging approaches are not appropriate for assessing spine structure is due to aspects of the incident and emitted light used to image them, which we will discuss briefly below.

While all the aforementioned methods are suitable for dendritic morphology reconstructions, additional considerations need to be made when imaging small structures such as dendritic spines (Table 1). The current gold-standard to spatially resolve small structures in biological samples is electron microscopy, as this allows image resolution on subnanometer scales. However, due to many factors in the preparation, imaging, and analysis of electron micrographic images, this does not generally scale well for large-scale longitudinal or transgenic studies due to the required number of biological replicates (see below).

**Table 1:** Cost/Benefit Analysis of Imaging and Cell Labeling Approaches Discussed^a^

method	pros	cons	cost	time
Golgi impregnation	• Efficient	• No cell specificity	Cheap	Rapid
	• Any species/strain	• Limited multiplexing		
	• High contrast			
	• White light visible	• Many cells labeled		
Fluorescent reporters	• Efficient	• Multiple animal line breeding	Less cheap	Rapid (once set up)
	• Cell-type specific			
	• Area/region specific	• Surgical techniques		
	• Can be multiplexed	• “Leaky” expression		
	• Super-resolution	• Many cells labeled		
Dye electrophoresis	• Efficient	• “Blind” approach	Cheap	Rapid (once set up)
	• Any species/strain	• Limited multiplexing		
	• Single cell resolution	• Skilled technique		
	• Super-resolution	• Requires filling rig		
*In utero* electroporation	• Efficient	• Surgical techniques	Less cheap	Less rapid
	• Cell-type specific	• Specialist equipment		
	• Area/region specific			
	• Can be multiplexed	• Many cells labeled		
	• Super-resolution			
Patch clamp	• Any species/strain	• Skilled technique	More expensive	Slow
	• Single cell resolution	• Specialist equipment		
	• Correlated physiology	• Low cell yield		
	• Transcriptomics			
	• Easily multiplexed			
	• Super-resolution			
Patch clamp + multiphoton	• Any species/strain	• Highly skilled	Very expensive	Slowest
	• Single cell/spine resolution	• Requires setup		
	• Correlated physiology	• Very low cell yield		
	• Transcriptomics			
	• Easily multiplexed			
	• Super-resolution			

a Methods are shown with their specific pros and cons, stated with their relative cost and time required.

The ease of preparation of fluorescent imaging samples and the ready availability of high-resolution light microscopes have greatly expedited the collection of such data with sufficient efficiency to sample many animals rapidly. However, this brings a major consideration to the table: the diffraction limit of light and how this may influence spine imaging. This diffraction limit of light, as defined by Ernest Abbe, sets the maximum resolution potential of a given wavelength of light, proportional to the wavelength of incident light and the numerical aperture (NA) of the objective lens (currently the best NA ∼ 1.5). For typical imaging using a green fluorophore (e.g., GFP), the smallest structure which can be faithfully resolved would be ∼250 nm. The identification and measurement of small structures can be improved with image processing (i.e., filtering, deconvolution, centroid analysis); however this is still limited to ∼120 nm resolution in the two-dimensional plane.^62^ In cortical pyramidal cells, dendritic spines are typically 1.5 ± 0.5 μm long (SD, range of 0.46–3.3 μm), with a shaft thickness of 0.23 ± 0.09 μm (SD, range of 0.07–0.50 μm), with an average head diameter of 0.59 ± 0.22 μm (SD, range of 0.23–1.30 μm), when analyzed using electron microscopy.^63^ This leads to a major confound when considering how to measure spine morphology, as the best spatial resolution using standard confocal microscopes one can achieve is ∼120 nm, which will lead to drastic overestimation of spine structure.^64,38^ This diffraction limit can be overcome with super-resolution imaging, such as stimulated emission depletion (STED) imaging, which can resolve a point of ∼25 nm using visible light^64,65^ and lower if combined with deconvolution and image postprocessing methods.^66^ While standard confocal imaging with deconvolution is sufficient to count spine numbers of dendritic shafts, measurements of spine structure can only be reliably assessed using approaches that overcome the diffraction limit of light. A recent advance that may make such morphological analysis more achievable to research groups without access to STED microscopes is the use of expansion microscopy, whereby tissue is embedded within a Matrigel, then physically expanded.^67^ Such techniques overcome the diffraction limit by changing the physical properties of the tissue itself, using readily available laboratory reagents, which can then be imaged using a standard confocal microscope. Indeed, they are particularly powerful when combined with immunohistochemical labeling for synaptic markers, such as postsynaptic density proteins, neurotransmitter receptors, and RNA molecules (reviewed in ref (68)). To date, however, few studies have employed expansion microscopy to measure dendritic spines in a high-throughput manner, although such morphological interrogation is feasible.^69^ Whether such techniques provide a more consistently reliable method to determine spine structure, such as in neurodevelopmental conditions, remains to be seen.

## How To Analyze Dendritic Spine Data

From an analytical perspective, there are several key elements that we may want to determine relating to dendritic spine properties: first, the number of dendritic spines, as a proxy for synapse number; second, what are the structural properties of those dendritic spines, to determine how electrically isolated they are from dendrites.^70^

To ascertain dendritic spine number, we are more likely considering their density, such as how many spines per unit dendritic length; very few researchers will actually count the total number of dendritic protrusions for each cell they label. Our view is that the most robust measurement of spine density can be achieved by (a) generating high-resolution, Nyquist sampled images of dendritic segments, (b) deconvolving those images to obtain a robust spatial profile of spines and dendrites, (c) faithfully counting all processes that emerge from those dendritic segments. This approach ensures that all processes that emerge from the dendritic shaft are recorded. There are, however, several key considerations that must be considered for this analysis. First is ensuring that collected images are an appropriate resolution for detection and deconvolution. As we stated above, the diffraction limit of light is a major limiter to spine morphology measurements. But for spine density it is sufficient to perform diffraction-limited imaging combined with deconvolution. For these images, the pixel resolution needs to ensure adequate sampling of spine structures and differentiation of dendritic spines. Let us assume that the average spine head width is 1 μm and each spine is 1 μm apart. A pixel resolution of 500 nm/0.5 μm should allow differentiation of two spines, assuming they are in the same focal plane. As such, the bare minimum resolution we require is 250 nm (rule of 2). However, in reality, spines are organized in three dimensions around the dendritic shaft and greater pixel resolution should be considered when taking into account the axial-plane (*z*-axis). We normally aim for 140 nm pixel resolution, combined with 140 nm z-steps, which, following deconvolution, allows resolution of spines at the diffraction limit.

Dendritic spine morphological analysis is a multistep process involving various methods and algorithms. The workflow of dendritic spine imaging analysis generally consists of five steps: preprocessing of raw images, detection and segmentation of spines, quantification of morphological features, and determination of spine phenotypes.

**Figure 3:**
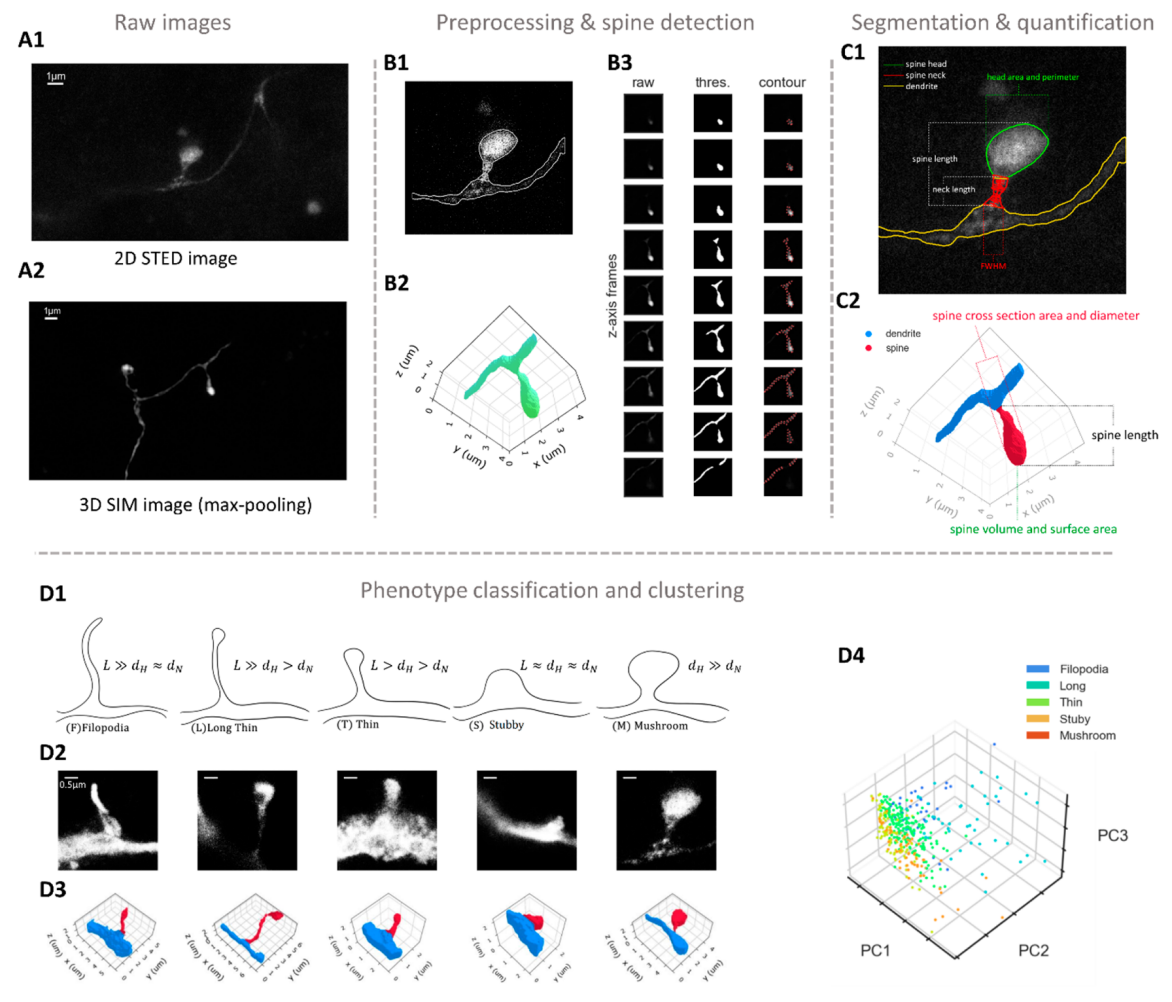
Dendritic spine data analysis workflow. (A) Example raw imaging data by 2D-STED (A1) and 3D-SIM (A2); scale bars, 1 μm. (B) Preprocessed and detected 2D-STED spine sample using wavelet filter and gradient field approach (B1), and 3D-SIM spine sample using multilevel thresholding and active contour approach (B2, B3). (C) Segmented and quantified 2D-STED spine sample (C1) and 3D-SIM spine sample (C2). (D) Commonly used spine phenotype categories (D1; *L*, spine length; *d*_H_, head diameter; *d*_N_, neck diameter); 2D-STED examples (D2; scale bars, 0.5 μm); 3D-SIM examples (D3); classified phenotype data set represented and visualized with PCA-based clustering (D4). Example data in this figure were acquired in mouse lateral superior olive. The source code for analyzing and plotting example 3D SIM data is available online (GitHub repository: https://github.com/libenzheng/dendritic_spine_processing_example).

Preprocessing is the first step in the analysis of dendritic spine imaging, and it involves commonly used processes such as cropping and filtering, as well as specifically developed methods to obtain 2D contours or 3D meshes of dendrites. For denoising purposes, raw images can be processed with median or unsharp mask filters and deconvoluted by using a sparse deconvolution algorithm^71^ or the Huygens deconvolution package (Scientific Volume Imaging, Netherlands). Additionally, for diffraction-limited imaging data, the resolution can be improved by using emerging machine learning approaches such as the effective subpixel convolutional neural network.^72,73^ For 2D STED imaging data (Figure 3A1), Levet et al. proposed a workflow to extract spine contour (Figure 3B1) that uses a wavelet filter to compute isolated spine head contours and a gradient field to reconnect the spine head to the dendrite shaft, allowing for the estimation of the spine neck contour.^74^ For 3D structural illuminated microscopy (SIM) imaging data (Figure 3A2), interpolation along the *z*-axis is an important step in preprocessing to compensate for the lower axial resolution in image acquisition. Kashiwagi et al. proposed a method for converting imaging data to 3D meshes (Figure 3B2) by utilizing the marching cube algorithm on binarized image voxels generated by multilevel Otsu thresholding and active contour models (Figure 3B3).^75^

Detection of dendritic spines in microscopy images can be a labor-intensive task, traditionally done manually by human operators. However, the use of automatic and semiautomatic detection approaches can increase the throughput of the process. One common strategy for detecting spines is to use the skeletonized dendrites, which has been implemented in both 2D (iterative deletion;^76,77^ Delaunay triangulation^74^) and 3D data (centerline^78^). Another strategy is to detect spines on the boundary of the dendrite shaft, which has been implemented using wavelet filters on 2D data,^79^ and elliptic cylinder fitting on 3D data.^75^ Additionally, spine detection can also be conducted automatically using multiscale opening algorithms^80,81^ and deep learning CNN-based methods.^72,73^

The segmentation of detected spines from the dendritic shaft can be achieved using similar strategies to those employed in spine detection. Methods such as skeletonization,^76^ dendrite shaft fitting,^75^ and multiscale opening algorithms^80^ have been utilized to approximate the spine base. The spine head and spine neck can then be further segmented through the use of Delaunay triangulation.^74^ Further various spine detection and segmentation methods have been comprehensively reviewed previously.^82^

Primitive morphological features of segmented dendritic spine samples can be directly computed, e.g., spine length, head area, and head volume. In the case of 2D STED spines (Figure 3C1), ellipse fitting on the spine head can provide measures such as head width, length, and aspect ratio.^83^ The width of the spine neck can be estimated by computing the full width at half-maximum (fwhm) of sampled neck sections.^74^ For 3D SIM spines (Figure 3C2), the shape of the spine head can be analyzed through the use of ellipsoid fitting or section ellipse fitting,^84^ which can also be used to determine the width of the neck. The surface curvature of the spine head can be represented by metrics such as the convex hull ratio and Gaussian curvature.^75^ Additionally, the synaptic area can be calculated based on further surface curvature analysis on manually selected synaptic regions.^84^

Quantified spines can be classified into several phenotypes based on their morphological features. Widely used phenotypes include mushroom, stubby, thin, long, and filopodia for longer developing spines (Figure 3D1–D3). Despite some extant evidence that synaptic strength is correlated to spine size^85,86^ and spine phenotypes,^87^ the physiological relevance and variability of the spine phenotype classification are still under investigation. Conventional methods for phenotype classification involve manual inspection or computation of width metrics^88^ and length metrics.^80^ Meanwhile, with the use of labeled training data sets, machine learning approaches have been shown to achieve comparable accuracy to human operators.^75,89^ Additionally, recent studies have suggested using clustering methods to automatically group spines with similar structural morphology rather than using predefined phenotype categories.^43,44,90^ This approach typically involves using principal component analysis (PCA) for dimensionality reduction and applying clustering algorithms (e.g., K-means and hierarchical clustering) on principal components to assign spine samples into clustered phenotype classes^75,84,90^ (Figure 3D4).

In contrast to morphology-based spine classification and analyses, an approach centered on fluorescent intensity had been implemented in multiple studies.^2,43,91,92^ In this approach, by leveraging the monotonic relationship between GFP brightness and single spine volume,^93^ the spine volumes were estimated by calculating the total integrated brightness (TIB) normalized to the adjacent dendritic shaft brightness. This approach offers a simple TIB metric for classifying spines and correlating them with physiological functions and dynamics and could circumvent the potential ambiguity associated with the uncertain morphological-function relationships across different spine phenotypes.

Once the morphological features and phenotypes of dendritic spines have been extracted and assigned, the samples are ready for the subsequent statistical analysis. There are various toolkits available for dendritic spine imaging analysis, such as SpineJ, an ImageJ plugin for quantifying 2D STED data,^74^ and DXplorer, a unifying spine analysis framework for 3D SIM data,^84,54^ and so on. Commercial imaging software packages, e.g., Imaris Filament Tracer (Bitplane, Oxford Instruments, UK), are also available for detecting and quantifying 3D dendritic spines,^94^ albeit accompanied by an increased expense. These tools allow for interactive conduct of the aforementioned steps and facilitate the interpretation of the results.

## Interpretive Considerations for Image Analysis Data

So far, we have discussed the technical considerations for morphological assay of cells in neurodevelopmental conditions. However, arguably the most important consideration is how you compare these findings between genotypes and across life. This should not be an afterthought (although that is sometimes easier said than done), but the planned experimental design should be established *a priori*. Indeed, many funding bodies (e.g., UK Research and Innovation, National Institutes of Health, European Research Council) require experimental design and power calculations before research is funded and should be followed. The best experiments start with a clear, testable hypothesis, e.g., gene X alters synapse number in such a way as to impair behavior. From this hypothesis, it is then possible to establish the most appropriate experimental plan, how you will statistically test this, and what criteria you will accept as inferring biological meaning. Indeed, once decided, the experimental plan should incorporate power calculation to determine an appropriate sample size for each group to be tested. For spine density, there is a wealth of resources that have previously determined observed spine densities for wild-type animals (e.g., hippocampal neurons^95^). These baseline data and variance can be incorporated into previous studies examining spine density to obtain a realistic estimate of effect size and thus the required sample size to affirm or reject the null hypothesis. Once these numbers are reached experimentally, further data collection should be halted as this risks overpowering the data set (P-hacking^96^).

**Figure 4:**
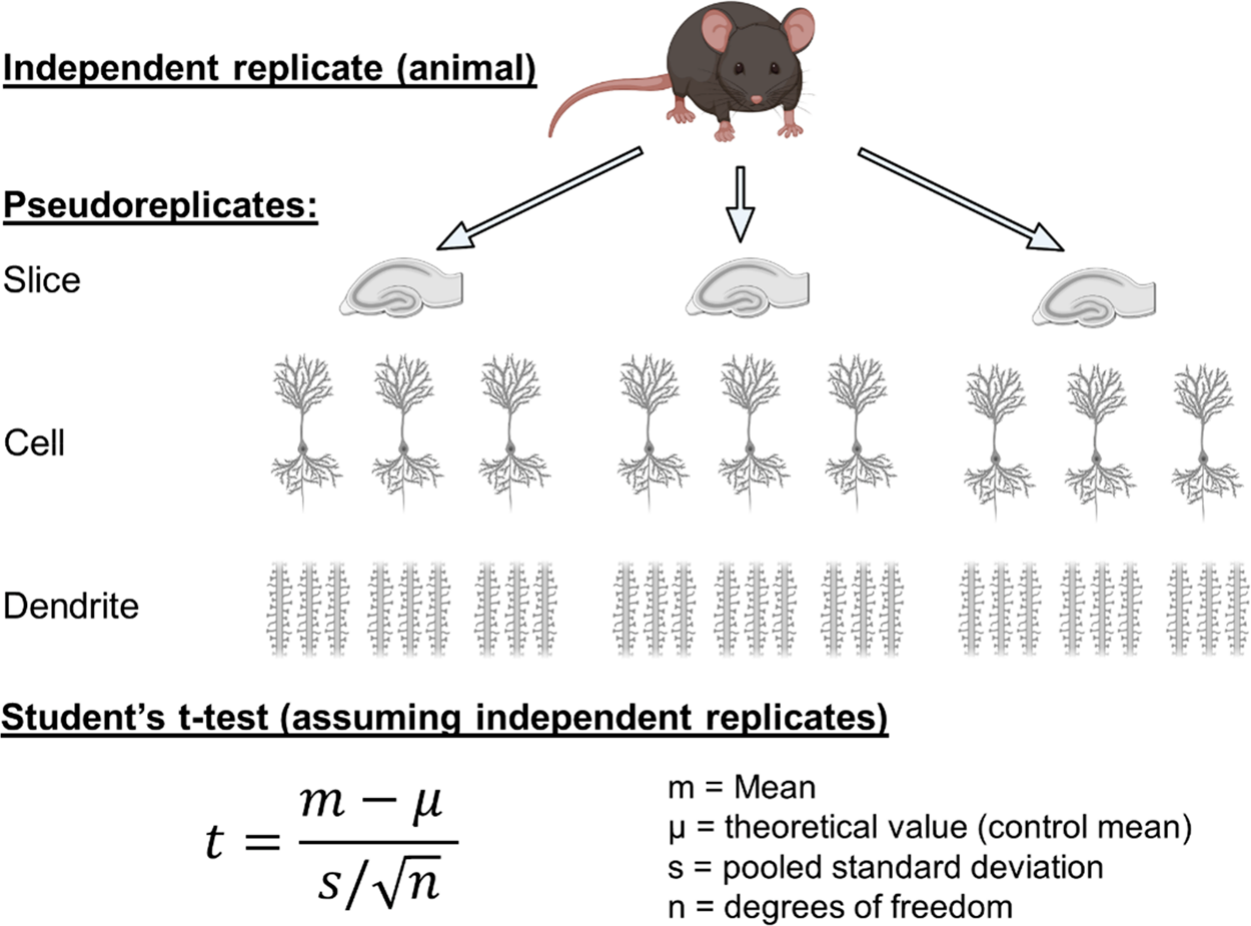
Pseudoreplication as a factor in spine imaging/analysis studies. Schematic representation of pseudoreplication as a product of repeated spine measurements from multiple dendrites, in multiple cells, from a single independent replicate (e.g., a mouse). Lower, the most common statistical test for comparing two independent groups: the Student’s *t* test. This test relies on independence of replicates. Note how artificially conflating the number of replicates can drastically increase the denominator when generating *t*-values.

These seemingly simple principles, however, assume that you have chosen your replicate appropriately in the first instance. But what is the most appropriate replicate? This raises the somewhat thorny subject of pseudoreplication (Figure 4A), by which we mean the inappropriate choice of replicate that overpowers the statistical analysis and leads to rejection of the null-hypothesis (a type 1 statistical error). The reason this is so manifestly important in image analysis is that biological effects can be small and transient and show a high degree of interanimal variability. As such, oversampling from a given biological replicate may lead to overt miscalculations of statistical significance, despite a very modest effect size (Figure 4A). This is particularly pertinent when using a conventional two-sample test (e.g., Student’s two-tailed *t* test) for analysis. Implicitly, within the formulas for many statistical tests like the *t* test is the requirement for the number of replicates included in the data sets (Figure 4B). If non-independent replicates, such as individual spines, dendritic segments, or cells from the same animal are used, there is an inherent sampling bias to these data and, as such, a drastic overpowering of these data.^97^ This overpowering inherently favors the generation of unfeasibly high numbers of replicates, resulting in the generation of high *t*-values, which in turn lead to the calculation of very low *p*-values, thus inappropriately rejecting the null hypothesis. For the vast majority of data sets, there are two ways to overcome such risks: either pool all measurements from a given cell or dendrite type for each animal or biological replicate or generate a more complete statistical model that takes into account intra- and interanimal variability and varying number of measurements per animal. These replicate choices apply equally to all types of data, regardless of the complexity of experiment, whether that be an *in vivo* imaging or *in vitro* primary cell culture study. Without robust, transparent experimental design and statistical analysis, we are perpetuating the issues of scientific reproducibility and ultimately increasing the number of experimental subjects required to support hypotheses.^98^

Animal average data have one distinct advantage in that you are accepting the idea that this considers the biological history of that individual (provided sampling is made from a homogeneous cell type or dendritic region). This can include aspects such as sensory/environmental experience, genetic diversity, sex, age, etc. This approach then allows fair comparison of truly independent replicates/samples that are required for classic statistical approaches such as Student's *t* test or ANOVAs. However, while this simplifies things from an interpretive perspective, it also risks removing key variability from within the replicate, which is far from perfect. A key drawback of this approach is when considering experiments with very low biological replicate yield (e.g., human induced pluripotent stem-cell lines or non-human primates), where it would be ethically or technically difficult to obtain a sufficiently high biological replicate count to warrant this type of data reduction. Nevertheless, clearly identifying the replicate used is critical, and these types of study favor more rigorous statistics. The second approach is to build a multicompartmental statistical model, such as a linear mixed-effects model. This approach lends itself to capturing the within subject variability and other off target sources of variability. By assigning these as random variables, they can be accounted for to reveal the variability and effect size that arise due to genotype and/or age. These when combined with *post-hoc* testing then allow for statistical comparison between groups. These approaches have been used by ourselves to great effect when tens to hundreds of repeated (nonindependent) measurements have been made from multiple animals.^14,15,55^ Nevertheless, these models still require sufficient biological and technical replicates to determine groupwise effects, which with respect to the approaches outlined above may require time-consuming and costly collection of multiple biological replicates. This is not a wasted venture, as such robustness of experimentation and analysis is the best way to ensure reproducibility of key findings.

Beyond statistical design aspects, a key consideration is the location of the dendrites that you are examining. Specifically, are they always measured in the same hierarchy, such as primary, secondary, or tertiary dendrites, as this will affect measured spine density and thus consistency of the resulting data set. Perhaps just as important is the afferent pathway with which those dendrites align, for example, a hippocampal pyramidal cell’s apical dendritic tuft will largely receive inputs from entorhinal cortex and possesses low synapse density; while oblique dendrites emerging from the apical dendrite align to Schaffer-collateral inputs and have a higher spine density,^95^ such dissection is critical if correlation with synaptic function is to be performed (see ref (99) and below). If the dendrite type is not routinely measured, this may introduce significant variability within the data, leading to possible inappropriate statistical inferences.

Ultimately though, the most robust method to infer biological meaning from such spine density or structural data is to confirm them functionally. This can require considerably more effort than that used to achieve the measurements of spine density in the first place. One particularly common method for such analysis is the use of electrophysiological methods, such as whole-cell patch-clamp recordings to measure spontaneous synaptic inputs to a given neuron. This approach has a significant benefit in that you can measure both functional and structural correlates of spine density in the same cells from the same animal. Combining these recordings with pharmacological blockage of voltage-gated sodium channels using tetrodotoxin allows for determination of the quantal synaptic properties of a given neuron.^100^ These can then be combined with methods, such as two-photon glutamate uncaging, to measure the activity of individual identified dendritic spines, and super-resolution imaging.^55^ While this latter study may be the extreme end of functional analysis, further studies have shown the importance of correlating spine structure and function.^101^

## Future Directions/Outlook

The development of new technologies in neuroscience and adjacent fields, such as molecular markers, reporter lines, and new imaging techniques, has allowed for many opportunities to further the dissection of function of spines,^102^ even to the level of the individual spine.^91^*In vivo* imaging of active dendrites (through isolating specific dendrites, dendrites engaged during behavior, or dendrites involved in *in vivo* plasticity paradigms and imaged with two-photon microscopy) can also be paired with anatomical characterization of the same dendrites in fixed processed tissue and analyzed further with STED, expansion microscopy,^67^ light sheet (with or without tissue clearing), or electron microscopy.^102^ These techniques have the advantage of combining fluorescent reporters (in many cases) with multiplex approaches such as multiplexed ion beam imaging^103^ or traditional immunolabeling approaches. Complex interactions in the nervous system can also be further explored with combining techniques, for example, recent work showing the relationship between microglia and synapses where electrophysiology, spine characteristics, and function of microglia were used together to provide a more holistic picture of brain function in schizophrenia.^2^ Further advances have arisen in the field of connectomics, employing either serial block-face imaging using light or electron microscopy to determine the structure and function of synaptic connections in local brain circuits.^55,104^ Combining complementary research tools will further enhance the rigor of research as well as allow experiments that were previously impossible to perform.

Many of the same considerations for dendritic spine analysis can be used when determining other types of anatomical questions related to developmental disorders. For example, measurement of myelin, which has recently been shown to be altered in autism spectrum disorders,^105−108^ can be performed using electron microscopy (EM) or immunofluorescent compatible techniques such as coherent anti-Stokes Raman scattering (CARS).^109,110^ Similar to considerations for dendritic spines, the technique used will likely depend on cost (EM being more expensive), with trade-offs for resolution on fine myelin microstructure (CARS being limited to the confocal microscope it is paired with), compatibility with dyes (CARS generally compatible with immunofluorescence depending on the experimental setup), or issues related to fixation or preparation requirements, in addition to feasibility such as availability of equipment (EM being more widely available across institutions). Therefore, similar considerations should be made when determining anatomical measurements across types of experiment, and the considerations presented here are widely applicable.

In conclusion, we highlight the varied technologies available for imaging of neuronal structures, with a particular focus on dendritic spines, which are biologically relevant functional units that are important in neurodevelopmental disorders and, being relatively small, have until recently posed challenges in imaging and quantification. We additionally provide some considerations regarding analyses pipelines and tools as well as issues concerning statistical testing. This Perspective will hopefully provide insight into which techniques are most useful and appropriate for future studies on anatomical measurements in neurodevelopmental disorders and more broadly the study of the nervous system.
